# Psychosis Assessment in Early-Stage Parkinson's Disease: Comparing Parkinson's Psychosis Questionnaire with the Brief Psychiatric Rating Scale in a Portuguese Sample

**DOI:** 10.1155/2012/469126

**Published:** 2012-05-28

**Authors:** I. Cargaleiro, M. Serra, J. Alves da Silva, B. Neto, M. Gonçalves-Pereira, P. Bugalho

**Affiliations:** ^1^Mental Health and Psychiatry Department, Hospital S. Francisco Xavier (CHLO), Estrada do Forte do Alto do Duque, 1495-005 Lisbon, Portugal; ^2^CEDOC and Department of Mental Health, Faculdade de Ciências Médicas, (FCM), Universidade Nova de Lisboa, (UNL), Campo dos Mártires da Pátria, 130, 1169-056 Lisbon, Portugal; ^3^Neurology Department, Hospital de Egas Moniz (CHLO), Rua da Junqueira 126, 1349-019 Lisbon, Portugal; ^4^CEDOC and Neurology Department, Faculdade de Ciências Médicas, (FCM) Universidade Nova de Lisboa (UNL), Campo dos Mártires da Pátria, 130, 1169-056 Lisbon, Portugal

## Abstract

Psychotic symptoms in Parkinson's disease (PD) are frequent, disabling, and an important prognostic factor. Thus, screening instruments for detecting psychosis in PD are needed. For this purpose, we applied the Parkinson's Psychosis Questionnaire (PPQ), a short structured questionnaire, which requires no specific training, along with the Brief Psychiatric Rating Scale, expanded version (BPRS-E), for rating general psychopathology, including psychotic symptoms. We evaluated, in a cross-sectional study, a Portuguese sample of 36 early-stage PD patients (mean age of 73 years; mean duration of illness of 3.2 years). The PPQ total score correlated with the BPRS-E total score (0.359; *P * = 0.032) and with the BPRS-E-positive symptoms score (0.469; *P * = 0.004). The prevalence of psychosis (41.7%) was higher than expected. Sampling bias and detection of minor psychotic phenomena may have contributed to this result. These findings suggest that the PPQ should be further evaluated as a feasible assessment for psychotic symptoms in PD.

## 1. Introduction

In the context of Parkinson's disease (PD), the term psychosis usually referred to a mental state characterized by hallucinations and/or delusions, occurring with a clear sensorium and a chronic course. In the past years, definitions have changed and the typical hallucinatory syndrome in PD now encompasses other related phenomena, such as minor phenomena, like illusions, sense of presence, and passage hallucinations [1].

Among the nonmotor features of Parkinson's disease (PD), psychotic symptoms are frequent, ranging from 20 to 30% of patients [2]. Over the course of PD, psychotic symptoms, once present, tend to be persistent and progressive [3]. The impact of psychosis is substantial in that it is associated with dementia, depression, earlier mortality, greater caregiver strain, and nursing home placement. Psychosis also has important treatment implications, as it limits the therapy of motor symptoms [4].

Recently, the Task Force of the Movement Disorder Society comprehensively reviewed the scales used to assess psychosis in PD [5]. Albeit none of the current scales has been shown to possess the necessary basic mechanistic and psychometric properties, it was suggested that, in the meantime, the selection of the current scales should be based on the goals of the assessment [5]. Therefore and aiming for a precocious detection of psychotic symptoms in PD, especially in early stage patients, we were interested in exploring easy-to-use instruments and we selected the Parkinson's Psychosis Questionnaire (PPQ) [2, 5]. It is a brief, structured instrument that was designed for screening psychotic symptoms in PD patients.

We set out to compare the use of a brief and structured screening tool for psychosis in PD, the PPQ, and of the Brief Psychiatric Rating Scale, expanded version (BPRS-E), as a gold standard [6].

## 2. Material and Methods

### 2.1. Study Type, Sample, and Location

The study had a cross-sectional design. The sample consisted of 36 consecutive patients (19 females and 17 males) with early stage PD, recruited from Hospital Egas Moniz Neurology Department's outpatient clinic (Lisbon, Portugal). None of the patients refused to enter the study.

To be included, patients were required to have less than 5 years of disease duration since the first motor symptoms were reported and a Hoehn and Yahr stage [7] from 1 to 2.5. Informed consent was obtained from all patients. Patients with diseases of the central nervous system other than PD were excluded from the study.

### 2.2. PD Diagnosis

PD was diagnosed by P.B., an experienced movement disorder specialist, according to validated clinical criteria [8].

### 2.3. Demographics and Clinical Variables

Patients' demographical data (gender, age, and education level) and clinical data (duration of illness, levodopa equivalent dose, Hoehn and Yahr stage) were collected. The method for converting the total daily dopaminergic therapeutic dose in levodopa equivalent dose was obtained from published formulas [9].

The evaluation of the patients' cognition was made using the Portuguese version of the Mini-Mental State Examination (MMSE) from Guerreiro et al. [10]. MMSE is a simple, widely used scale to detect cognitive impairment. Although there is a debate on its usefulness as a screening tool for cognitive impairment in PD [11], it may still be considered appropriate for this purpose [5, 12]. In addition, the Frontal Assessment Battery (FAB) was applied for the same purpose [10]. FAB is a rapid screening battery that has been validated for PD [13–15].

### 2.4. Evaluation of Psychosis/Psychotic Symptoms

Psychiatry trainees (I.C., M.S., J.A.S., and B.N.) assessed the patients for psychotic symptoms, using two different instruments: a structured and easy-to-administer questionnaire that specifically addresses psychotic and related symptoms in PD—the PPQ [2]—and a widely used general psychopathology semistructured instrument—the BPRS-E [6].

The PPQ was developed as a 14-item screening instrument for early recognition of psychosis in PD. The specificity and sensitivity reported by the developers of PPQ were 92.1% and 100%, respectively, using Structured Clinical Interview for DSM-IV-TR Axis I Disorders (SCID) as the gold standard [2]. This scale includes probe questions followed by detailed questions regarding four domains. The first domain includes three questions about the presence or absence of sleep disturbance. The following domain assesses, in four questions, the occurrence of hallucinations and/or illusions, including visual, auditory, and tactile hallucinations. The third domain includes five questions to detect different types of delusions: persecutory, jealousy, poisoning, abandonment, and control. The fourth domain assesses place and time orientation. Within a domain, any positive answer triggers inquiries about frequency (1–3 points) and severity (1–3 points). Each subscore is the product of the frequency multiplied by the severity score for that symptom domain. The total score is obtained by summing all subscores. PPQ caseness is defined by at least a positive score on the domains of hallucinations/illusions and/or delusions. We used our Portuguese translation of the PPQ. Two English-fluent medical doctors among the authors (BN, JAS) were responsible for this translation. Given the simple straightforward nature of the translation process, backtranslation procedures were skipped. Moreover, face and content validity of the original version are assumed to be preserved.

The BPRS-E [6, 16] was designed for measuring overall psychopathological change in patients with schizophrenia. Validity and reliability have been widely documented [6, 16, 17]. It is administered in a semistructured manner, and it takes about 30 minutes to complete. It comprehends 24 items that can be scored from 1 (not present) to 7 (very severe). The total BPRS-E score is the sum of the scores for each of the 24 items. A Portuguese translation (Caldas de Almeida, Gusmão, Talina, and Xavier—Universidade Nova de Lisboa, 1996; unpublished document) of the BPRS-E was used. Since there is no study describing the factor structure of BPRS-E for PD, we used a BPRS-E factor solution that was described in a sample of European patients with schizophrenia [17] and recently used in Portuguese research [18]. This factor solution includes four subscales: positive symptoms (grandiosity, suspiciousness, hallucinations, unusual thought content, and conceptual disorganisation), negative symptoms (disorientation, blunted affect, emotional withdrawal, motor retardation, self-neglect, uncooperativeness, mannerism, and posturing), manic excitement/disorganization items (hostility, elevated mood, bizarre behaviour, self-neglect, uncooperativeness, excitement, distractibility, motor hyperactivity, mannerism and posturing), and depression/anxiety components (anxiety, depression, suicidality, guilt content, somatic concern, and tension). The subscales score is the sum of the scores for each item within a subscale. In this paper, mean scores (1 to 7) are presented for total BPRS-E and subscale scores.

Assessments with the BPRS-E and PPQ were conducted by the same interviewer for each patient. However, to minimize the bias related to the lack of occultation, the semistructured instrument (BPRS) was applied first. Each psychiatric trainee evaluated approximately 25% of the sample.

### 2.5. Statistical Analysis

Due to small sample size and the lack of a normal distribution in most continuous variables, we chose to use a nonparametric statistical analysis. Mann-Whitney test was used to compare the mean values of the BPRS-E factors and levodopa equivalent dosages (LED) between PPQ cases and noncases. The Spearman's rank correlation coefficient was used to evaluate the association strength between LED and psychotic symptoms severity and also between PPQ scores and BPRS-E scores. The level of statistical significance was set at *P* ≤ 0.05.

All statistical analyses were performed using SPSS 18.0. (SPSS, Inc., Chicago, IL).

## 3. Results and Discussion

### 3.1. Results

The clinical and demographical characteristics of the 36 patients in the sample are summarized in Table 1.

Thirty-two patients were on dopaminergic treatment: 17 on levodopa only, 6 exclusively on dopaminergic agonists, and 9 on both types of drugs. Amantadine was not used and neither neuroleptic nor cholinesterase inhibitors.

Considering the whole sample, LED showed a correlation trend, although with a low level of evidence, with PPQ total score (*r*_*s*_ = 0.311; *P* = 0.065), and BPRS-E positive symptoms (*r*_*s*_ = 0.322; *P* = 0.056). There was no evidence of correlation between LED and the other BPRS-E factor scores, or between LED and the Total BPRS-E score.

Fifteen patients were defined as cases by the PPQ. Of these, 4 reported the presence of illusions but not of major psychotic phenomena. In fact, as these psychotic symptoms were not disturbing to the patients, they did not lead to any specific changes in the management of the patients.

Although the mean LED was higher in PPQ cases than in noncases, this difference was not statistically significant after correcting for multiple comparisons (Table 2). MMSE and FAB scores were similar between cases and noncases.

Mean scores of the BPRS subscales did not differ in PPQ cases and noncases, except for the positive symptom subscale scores, which were higher in the cases' group (Table 2).

The PPQ total score was significantly correlated with total BPRS-E and positive symptoms BPRS-E scores (Table 3).

### 3.2. Discussion

Prevalence of psychotic symptoms in PD is variable among different studies. Cross-sectional studies of clinical populations have reported prevalences as disparate as 25% [19] and 75% [20]. This may be due to differences regarding the type of psychotic phenomena assessed, different diagnostic criteria, and other methodological issues. Williams et al. [20], for instance, encompassed minor psychotic phenomena in their definition of psychosis and identified them in a large majority (72%) of their PD patients. However, this study was not limited to early stage patients.

In our study, minor psychotic phenomena were responsible for the definition of approximately one quarter of cases. This could help to explain the high prevalence of psychotic symptoms found in our sample of patients with early stage PD. Furthermore, Graham et al. [19] described that the proportion of hallucinations did not increase in a linear fashion with PD progression. They found that there was a peak of onset of perceptual disturbances during the first five years of disease. However, subsequent longitudinal studies did not confirm this finding [21].

A number of studies have also suggested that demographic factors, like age, could be related to a high prevalence of hallucinations in an early stage population, independent of disease duration [22, 23]. Actually, the majority of our patients were aged and that may have contributed to a higher prevalence of psychotic symptoms in our sample.

Sampling bias could have also contributed to these results: first, our sample was not randomized; second, one may argue that early PD patients with manifest psychotic symptoms are more easily referred to a neurology outpatient clinic.

LED was not significantly associated with psychotic symptoms in this sample. The relation of dopaminergic treatment with psychotic symptoms in PD is still a matter of debate. Data on untreated patients with early PD is scarce and conflicting [4]. Furthermore, several cross-sectional studies could not identify differences in LED between patients with and without hallucinations [4]. However, a recent meta-analysis [24] reported that dopamine agonists were associated with higher odds of experiencing hallucinations, when compared with both placebo and levodopa. Dopaminergic treatment may be an important risk factor for psychotic symptoms in PD, as hallucinations in drug-free PD patients are very rare. In fact, none of our drug free patients presented with hallucinations. We were not able, though, to evaluate the association of different treatment profiles with psychosis because of the small number of patients in each group (levodopa, dopaminergic agonists, or both).

A longitudinal study found that cognitive impairment in early PD predicted the development of psychotic symptoms with treatment [25]. In this cross-sectional scenario, we were not able to reproduce an association between cognitive scores and the presence of psychosis according to the PPQ case definition.

Albeit not above moderate levels, an association was found between the PPQ scores and the BPRS-E scores. Interestingly, this association was only evident concerning the BPRS-E total score and the positive symptoms subscale score, which includes the psychotic symptoms most often reported in PD. In fact, BPRS-E-positive symptoms would be expected to correspond *grosso modo *to the hallucination/illusion and delusional categories of the PPQ. Moreover, PPQ cases presented significantly higher scores of BPRS-E positive symptoms when compared to noncases. Therefore, there is an argument to further explore the use of the PPQ for PD psychosis both in research and clinical settings.

Along with selection bias, the main limitations of this study are the small sample size and the lack of a control group. Furthermore, our evaluation did not include follow-up data. Regarding assessments, we had no previous knowledge of the detailed psychometric properties of the PPQ in Portugal, and we postulated a BPRS-E factor structure, which in fact was not originated in PD populations. Also, since the BPRS-E is an instrument that provides a continuous measure of psychopathology and is not a diagnostic tool, we could not calculate PPQ sensitivity and specificity in this population. The fact that evaluations were not conducted blindly by the interviewers, who used the PPQ and the BPRS in the same patient, may have contributed to some bias. Nevertheless, by applying the PPQ systematically after the BPRS, we tried to reduce this bias. The structured nature of the PPQ would not allow for any probing that could have been prompted by the answers to the BPRS-E.

## 4. Conclusions

In this paper, we described a small nonrandomized sample of early stage PD Portuguese outpatients, exploring PPQ validity as related to BPRS-E results in the detection of PD psychosis.

We found an acceptable agreement between PPQ and BPRS-E assessments, and this supports, to some extent, the PPQ as a feasible and valid screening instrument for psychosis in PD, namely, in early stages of the disease. While the PPQ is easily used and allows for quick administration procedures, the BPRS-E remains a more demanding, semistructured instrument that requires specific training.

Easy and quick-to-use tools like PPQ, if valid in early PD, may contribute to a precocious identification of psychotic symptoms and hopefully to a better clinical management of patients with PD.

## Figures and Tables

**Table 1:** Sociodemographic and clinical data (*n* = 36).

Gender (male/female)	17/19
Age (years)	73.17 (6.54)*
Education	4 (4)*
Duration of illness (years)	3.17 (1.30)*
Levodopa equivalent dose (mg)	300** (range 0–1500)
Hoehn and Yahr stage (on)	2** (range 1–3)
MMSE	27.22 (2.53)*
FAB	13.31 (3.13)*
PPQ (total score)	3.69 (3.42)* (range 0–12)
PPQ (% cases)	41.7
BPRS (total score)^ +^	1.26 (0.16)*
BPRS (positive symptoms score)^ +^	1.24 (0.31)*
BPRS (negative symptoms score)^ +^	1.19 (0.24)*
BPRS (depression/anxiety score)^ +^	1.60 (0.54)*
BPRS (mania/hostility score)^+^	1.04 (0.05)*

*mean (standard deviation).

**median.

^+^weighted scores; range 1 (not present) through 7 (extremely severe).

**Table 2:** Comparison of levodopa equivalent dose and BPRS scores between PPQ cases and noncases.

	PPQ non-cases mean (standard deviation)	PPQ cases mean (standard deviation)	Adjusted^+^ *P* value (Mann-Whitney test)
Levodopa equivalent dose	302.38 (377.07)	527.00 (363.73)	0.336
MMSE	27.33 (2.9)	27.07 (1.9)	>1
FAB	11.67 (3.12)	10.80 (3.19)	>1
BPRS—total score	1.23 (0.16)	1.31 (0.16)	>1
BPRS—positive symptoms	1.10 (0.16)	1.43 (0.37)	**0.016**
BPRS—negative symptoms	1.15 (0.24)	1.24 (0.23)	0.936
BPRS—mania/disorganization	1.04 (0.06)	1.03 (0.051)	>1
BPRS—depression/anxiety	1.62 (0.57)	1.57 (0.51)	>1

^+^Bonferroni correction was used to correct for multiple comparisons.

**Table 3:** Correlations between PPQ total score and BPRS scores (total and subscale scores). Spearman's rank correlation coefficient was used (*n* = 36).

	PPQ total score
BPRS (total score)	**0.359**; **P** = 0.032
BPRS (positive symptoms score)	**0.469**; **P** = 0.004
BPRS (negative symptoms score)	0.124; *P* = 0.472
BPRS (depression/anxiety score)	0.205; *P* = 0.231
BPRS (mania/hostility score)	−0.109; *P* = 0.528
